# Epidemiology of visceral leishmaniasis in municipalities of Mato Grosso and the performance of surveillance activities: an updated investigation

**DOI:** 10.1590/S1984-29612024008

**Published:** 2024-02-05

**Authors:** Jaqueline Aparecida Menegatti, Álvaro Felipe de Lima Ruy Dias

**Affiliations:** 1 Programa de Pós-Graduação Stricto Sensu em Biociência Animal, Faculdade de Medicina Veterinária, Universidade de Cuiabá - UNIC, Cuiabá, MT, Brasil; 2 Laboratório Central de Saúde Pública - Lacen, Secretaria de Estado de Saúde, Cuiabá, MT, Brasil

**Keywords:** Protozoan, sand fly, zoonosis, Brazil, protozoário, flebotomíneo, zoonose, Brasil

## Abstract

Visceral leishmaniasis (VL) is considered a globally neglected disease. To address the problem of VL endemic to Brazil, the Visceral Leishmaniasis Control Program (VLCP) was created, which recommends the development of health surveillance actions such as the identification of human and canine cases, vector control and prevention of disease. We aimed to investigate the epidemiological situation of VL in municipalities of the State of Mato Grosso (MT) and assess the execution of VLCP activities. Data on human cases were obtained from the Information System for Notifiable Diseases (SINAN), and data from entomological and canine inquiry were provided by the State’s Health Department. Analyzes from the period 2019 - 2021 recorded 30 cases of human VL, distributed among 16 municipalities. Vectors were identified in 50% of the municipalities where entomological investigations were carried out, and the predominant specie was *Lutzomyia longipalpis*. A total of 15,585 dogs were subjected to serological examination, of which 18.91% tested seropositive for *Leishmania infantum*. However, it must be emphasized that only three municipalities conducted consecutive inquiries involving canine VL. Although VL is distributed widely throughout the State, only a few municipalities have undertaken the actions of the VLCP, thus highlighting the neglected status of the disease.

## Resumo

A leishmaniose visceral (LV) é considerada uma doença mundialmente negligenciada. Em resposta ao cenário endêmico da LV no Brasil, foi criado o Programa de Controle da Leishmaniose Visceral (PCLV), que preconiza o desenvolvimento de ações de vigilância em saúde, como a identificação de casos humanos e caninos, controle vetorial e prevenção da doença. Objetivamos investigar a situação epidemiológica da LV nos municípios de Mato Grosso (MT), e avaliar se as atividades do PCLV estão sendo executadas. Os dados de casos humanos foram obtidos no Sistema de Informação de Agravos de Notificação (SINAN). E os dados dos levantamentos entomológicos e caninos foram disponibilizados pela Secretaria de Estado de Saúde. As análises do último triênio (2019 - 2021) registraram 30 casos de LV humana, distribuídos entre 16 municípios. Vetores foram identificados em 50% dos municípios que conduziram o levantamento entomológico, e a espécie predominante foi *Lutzomya longipalpis*. Um total de 15,585 cães foram submetidos a exame sorológico. Destes, 18,91% foram sororreagentes para *Leishmania infantum*. No entanto, é importante ressaltar que somente três municípios conduziram inquérito canino de forma consecutiva. A LV apresenta ampla distribuição no Estado, mas poucos municípios executaram as ações do PCLV, evidenciando a situação negligenciada da doença.

## Introduction 

Leishmaniasis is considered a neglected disease of significant worldwide relevance, and its most severe form is Visceral Leishmaniasis (VL), characterized as chronic, systemic, and with a high lethality rate (WHO, 2023). The agents responsible for this disease are intracellular protozoa belonging to the family Trypanosomatidae, genus *Leishmania*. In the Americas, the causative species is *Leishmania infantum* (Oliveira et al., 2018; Donato et al., 2020; OPAS, 2022).

In 2018, 92 countries were considered endemic for leishmaniasis, encompassing a population of over one billion individuals susceptible to infection. It is estimated that around 30,000 new cases of VL occur annually worldwide (WHO, 2023). An average of 3,379 cases per year occurred in Brazil in the last 10 years, distributed in 21 of the country’s 27 States (Brasil, 2021). The State of Mato Grosso (MT) is considered endemic for VL, with annual reports of cases in humans and canines in 43.26% (61/141) and 68.79% (97/141) of its municipalities, respectively (Mato Grosso, 2023).

The disease continues to expand widely due to the adaptation of the sandfly species *Lutzomyia longipalpis* and *Lutzomyia cruzi* to the urban environment (Brasil, 2021). Additionally, canines, rodents and marsupials are regarded as protozoan reservoirs. Among them, dogs are considered the main urban reservoirs due to their close relationship with humans and their high cutaneous parasitemia, which facilitates vector infection (Dias et al., 2020; Brasil, 2021).

VL prevails in human populations living in precarious socio-economic conditions, and affects mainly children under five years old and patients with comorbidities (Donato et al., 2020; OPAS, 2022). In this context, health surveillance plays a pivotal role in identifying transmission or risk areas, especially in regions without a history of human or canine cases of the disease. This approach aims to minimize or prevent new cases. It should be noted that leishmaniasis control strategies are adapted to specific regional characteristics. However, it is imperative for control strategies to be implemented in an integrated manner, encompassing health education initiatives, environmental management, as well as vector and canine reservoir control measures (Brasil, 2021).

Thus, the objective of this study is to contribute with current information about the epidemiological situation of VL in the municipalities of the State of MT and assess whether surveillance activities of the Visceral Leishmaniasis Control Program (VLCP) are being carried out by the municipalities in this State.

## Materials and Methods

Mato Grosso has 141 municipalities, all located in the Legal Amazon region, in Central-West Brazil, and a population of 3,567,234. The State capital, Cuiabá, is located in the geographic center of South America.

The data on autochthonous cases of human VL (HVL), as well as entomological and canine inquiries carried out from 2019 to 2021, were obtained from the Information System for Notifiable Diseases (SINAN), which are available on the website of the State Health Department (SES) of Mato Grosso (Mato Grosso, 2023).

Human data were expressed by the HVL incidence coefficient (number of autochthonous HVL cases x 100,000 inhabitants/population), as recommended by the Ministry of Health (MS) (Brasil, 2021). Entomological investigations were carried out using light traps recommended by the Centers for Disease Control and Prevention (CDC), which were installed for three consecutive nights in the peridomicile of pre-selected houses, and the data resulting from the identification of sandflies were expressed in absolute numbers. Canine VL (CVL) cases were identified through canine epidemiological investigations and spontaneous inquiries. The diagnostic method consisted of immunochromatographic screening tests (TR DPP®) and the enzyme immunoassay (EIA) served as the confirmatory test. The number of reactive dogs was calculated based on the ratio of reactive samples to the number of collected samples.

## Results

From 2019 to 2021, 30 autochthonous cases of HVL were recorded in the State of MT, distributed among 16 municipalities, 90% (27/30) of which came from urban areas. During this period, the highest disease incidence rate was observed in the municipality of Denise, although most cases are concentrated in Rondonópolis (Table 1). Six cases with indeterminate infection locations were recorded, along with one case imported from Sonora, in the State of Mato Grosso do Sul. Albeit present in the database, these cases were not included in the analysis.

**Table 1:** Occurrence of human cases of visceral leishmaniasis in municipalities in the state of Mato Grosso, Brazil, from January 2019 to December 2021.

**Year**	**Municipalities**	**Cases**	**Incidence**	**Gender**		**Residence location**	
				**Male Female**		**Urban Rural**	
2019	Alto Paraguai	1	8.8	1	-	-	1
	Colíder	1	3.0	-	1	1	-
	Confresa	1	3.2	1	-	1	-
	Nova Olímpia	1	4.9	1	-	1	-
	Denise	1	10.6	1	-	1	-
	Cuiabá	1	0.2	1	-	1	-
	N. Sra. do Livramento	1	7.6	1	-	1	-
	Rondonópolis	2	0.9	-	2	2	-
	Total	9	0.3	6	3	8	1
2020	Alto Taquari	1	9.2	-	1	1	-
	Barra do Garças	1	1.6	1	-	1	-
	Canarana	1	4.6	1	-	1	-
	Confresa	1	3.2	1	-	1	-
	Primavera do Leste	1	1.6	-	1	1	-
	Rondonópolis	7	3.0	5	2	6	1
	Chapada dos Guimarães	1	5.1	1	-	-	-
	Total	13	0.4	9	4	12	1
2021	Lucas Do Rio Verde	1	1.5	-	1	1	-
	Nobres	1	6.5	1	-	-	1
	Rondonópolis	5	2.1	5	-	5	-
	Várzea Grande	1	0.4	1		1	-
	Total	8	0.2	7	1	7	1

-: indicates no result.

Among the autochthonous cases, 20% (6/30) occurred in the age group of 1 to 4 years, 23.33% (7/30) in the age group of 29 to 49 years, 36.66% (11/30) in the age group of 40 to 59 years, and 10% (3/30) in the age group of 59 years. The other cases had a single case per age group: under 1 year, 5 to 9 years and 10 to 19 years.

Only seven of the 16 municipalities with HVL cases carried out just one entomological investigation during the triennium. In total, entomological investigations were conducted on at least one occasion in 11.34% (16/141) of the municipalities. Of these, VL vectors were identified in 50% (8/16) of the cases. However, the isolated presence of the *Lutzomyia cruzi* species was also recorded in one municipality, and both vector species were identified in three municipalities (Table 2).

**Table 2:** Entomological surveys carried out in the municipalities of the state of Mato Grosso, Brazil, from January 2019 to December 2021.

**Municipalities**	**2019**	**2020**	**2021**
Chapada dos Guimarães	-	**-**	Negativo
Cláudia	Negativo	-	-
Confresa	*L. longipalpis*	**-**	**-**
Cuiabá	*L. longipalpis*	**-**	**-**
Juara	*-*	Negativo	**-**
Nobres	*L. longipalpis/cruzi*	**-**	**-**
Nossa Senhora do Livramento	*L. longipalpis/cruz*	**-**	**-**
Nova Canãa do Norte	Negativo	-	-
Pedra Preta	*L. cruzi*	**-**	**-**
Peixoto de Azevedo	*L. longipalpis/cruzi*	**-**	**-**
Rondonópolis	*-*	**-**	**-**
Santo Antônio do Leverger	*-*	*-*	*L. longipalpis*
São José do Povo	Negativo	**-**	**-**
Sinop	Negativo	Negativo	Negativo
Tesouro	*-*	-	-
Várzea Grande	*L. longipalpis*	**-**	**-**

-: indicates no result.

Canine serological investigations were conducted in 30.49% (43/141) of the municipalities during the triennium, i.e., 13.47% (19) in the first year, 12.05% (17) in the second year, and 19.85% (28) in the third year. During the period under evaluation, 53.48% (23/43) of the municipalities presented reactive results for CVL. However, among these 43 municipalities, only 12 (27.9%) collected the number of samples established by the VLCP, and Poconé was the only municipality that conducted the canine survey for three consecutive years (Table 3).

**Table 3:** Canine visceral leishmaniasis survey carried out in the municipalities of Mato Grosso, Brazil, from January 2019 to December 2021.

**Municipalities**	**2019/Sampled**		**2020/ Sampled**		**2021/ Sampled**	
	**C**	**R%**	**C**	**R%**	**C**	**R%**
Alto Araguaia	0	-	0	-	74	8.10%
Alto Garças	24	-	0	-	114*	1.75%
Alto Taquari	0	-	0	-	50	2%
Araguaiana	0	-	30	-	0	-
Barra do Garças	0	-	614	8.96%	823	7.47%
Cana Brava do Norte	0	-	19	-	0	-
Colíder	24	-	1	-	0	-
Campo Verde	0	-	0	-	91	12.08%
Chapada dos Guimarães	0	-	0	-	66*	21.21%
Confresa	0	-	3	-	0	-
Cuiabá	1,136*	10.73%	414	15.07%	455	25.71%
Feliz Natal	0	-	8	-	0	-
General Carneiro	0	-	80*	-	37	-
Itaubá	0	-	15	-	0	-
Itiquira	0	-	0	-	71	4.22%
Jaciara	321	29.90%	0	-	428	24.29%
Juara	20	-	0	-	80*	-
Juína	0	-	0	-	58*	-
Juscimeira	121	3.30%	0	-	160*	11.25%
Lucas do Rio Verde	0	-	13	7.69%	0	-
Marcelândia	1	-	0	-	0	-
Mirassol D’Oeste	0	-	0	-	25	12%
Nobres	0	-	0	-	160	6.25%
N. Sra. do Livramento	80	8.75%	0	-	71	19.71%
Nova Canaã do Norte	4	-	10	-	0	-
Nova Guarita	20	-	0	-	0	-
Nova Mutum	0	-	4	-	0	-
Nova Xavantina	0	-	36	-	0	-
Novo Horizonte do Norte	20	-	0	-	58*	-
Peixoto de Azevedo	249*	1.20%	0	-	0	-
Planalto da Serra	50*	6%	0	-	48	-
Poconé	111*	23.42%	63*	31.74	72*	26.38%
Pontal do Araguaia	0	-	251*	8.36%	201*	15.42%
Ponte Branca	0	-	0	-	40	-
Porto dos Gaúchos	20	-	0	-	40	-
Poxoréo	448	12.5%	0	-	943	17.70%
Rondonópolis	172	31.39%	0	-	1	-
São Pedro da Cipa	0	-	0	-	1	-
Sinop	0	-	5	-	154	0.62
Tabaporã	20	-	0	-	0	-
Terra Nova do Norte	0	-	0	-	20	-
Torixoréu	0	-	66*	1.51%	38	2.63%
Várzea Grande	210	-	45	15.55%	0	-
Total	3,051	12.09%	1,677	10.13%	4,379	13.24%

C: Collected; R%: percentages of reagent samples; -: indicates no result.

* Municipalities that reached the sampling target.

Canine investigations and spontaneous inquiries occurred in only 18.43% (26) of the municipalities in MT during the triennium (Table 4). The serological investigations resulted in a total of 15,585 samples collected between 2019 and 2021, 18.91% (2,948) of which were reactive to CVL.

**Table 4:** Results of the spontaneous inquiries for canine visceral leishmaniasis in the municipalities of the state of Mato Grosso, Brazil, from January 2019 to December 2021.

**Municipalities**	**2019/Sample**		**2020/ Sample**		**2021/ Sample**	
	**C**	**R%**	**C**	**R%**	**C**	**R%**
Alto Araguaia	6	33.33%	0	-	28	7.14%
Alto Garças	16	-	0	-	1	-
Alto Taquari	1	100%	0	-	0	-
Apiacás	0	-	1	-	0	-
Barão de Melgaço	0	-	0	-	3	-
Barra do Garças	0	-	438	34.47%	409	33.74%
Campo Verde	25	8%	0	-	3	-
Chapada dos Guimarães	0	-	0	-	6	16.66%
Cláudia	27	-	35	-	38	-
Cuiabá	407	43.98%	339	53.39%	473	54.33%
Dom Aquino	1	-	0	-	0	-
Feliz Natal	36	-	32	-	32	-
Guarantã do norte	0	-	0	-	4	-
Guiratinga	69	27.53%	0	-	141	26.95%
Ipiranga do Norte	9	-	9	-	20	-
Itanhangá	4	-	3	-	20	10%
Itiquira	26	-	0	-	45	24.44%
Jaciara	1	-	0	-	0	-
Juscimeira	3	33.33%	0	-	0	-
Lucas do Rio Verde	29	10.34%	2	100%	5	-
Mirassol D’oeste	0	-	6	16.66%	0	-
Nobres	0	-	0	-	13	46.15%
Nova Mutum	63	20.63%	143	1.43%	100	8.0%
Nova Ubiratan	7	-	10	-	50	-
Novo Horizonte do Norte	0	-	0	-	58	-
Peixoto de Azevedo	0	-	20	-	0	-
Pedra Preta	131	39.69%	0	-	128	48.43%
Planalto da Serra	14	21.42%	0	-	0	-
Poconé	63	25.39%	11	36.36%	19	-
Porto dos Gaúchos	13	61.63%	0	-	178	50%
Primavera do Leste	46	17.39%	0	-	67	20.89%
Ribeirãozinho	0	-	10	-	0	-
Rondonópolis	333	56.75%	0	-	118	55.084%
Santa Carmen	40	-	0	-	16	-
Santa Rita do Trivelato	9	11.11%	3	-	9	-
São José do Povo	100	59.00%	0	-	0	-
São Pedro da Cipa	2	-	0	-	35	-
Santo Antônio do Leste	4	-	0	-	0	-
SINOP	88	-	184	1.08%	39	12.82%
Sorriso	29	6.89%	114	-	142	-
Tabaporã	0	-	0	-	20	-
Tapurah	12	-	76	-	31	-
Terra Nova do Norte	0	-	0	-	1	-
Tesouro	0	-	0	-	36	22.22%
Torixoréu	0	-	2	-	0	-
União do Sul	25	-	40	-	40	7.5%
Várzea Grande	272	9.92%	166	21.68%	463	29.80%
Vera	36	-	31	-	65	-
Total	1,947	30.04%	1,675	23.70%	2,856	29.65%

C: Collected; R%: percentages of reagent samples; -: indicates no result.

## Discussion

Up until the 1980s, VL predominated in rural areas (Abreu et al., 2021). However, new epidemiological patterns emerged due to various factors that facilitated the geographical relocation of vectors and reservoirs from their native regions. These changes include the dispersion and adaptation of vectors and reservoirs to the urban environment, deforestation, migration, and urbanization (Osaki et al., 2021). Additionally, the adaptation of *Lutzomyia longipalpis* to the anthropized environment has allowed the maintenance of the rural transmission cycle and the dissemination of VL in urban areas (Rangel & Vilela, 2008).

As a result of the endemic situation of VL in Brazil, the VLCP was created to reduce dissemination of the disease through the diagnosis and treatment of human cases, lower the sandfly population, and control infected dogs. The effectiveness of these measures depends on the health surveillance of municipalities, which should include the identification of transmission areas, assessment of vector distribution, adequate medical care and monitoring of dogs (Brasil, 2021). These actions should be based on epidemiological and entomological studies, enabling the identification of risk areas and the planning of control activities (Carvalho et al., 2022).

In this context, the analysis of data between 2019 and 2021 in MT revealed gaps in information collection. Only seven of the 16 municipalities with human case records conducted entomological investigations, and only one conducted a canine inquiry, as recommended by the VLCP. This lack of adequate inquiry can hinder effective monitoring of the disease and facilitate its spread to other regions.

The findings of our investigation revealed the occurrence of autochthonous cases of HVL in five new municipalities in the State: Alto Taquari, Colíder, Denise, Lucas do Rio Verde and Nova Olímpia. The relevance of these findings stems from the unprecedented nature of these records, indicating a recent spread of the disease, and from the constant notification of autochthonous cases of VL in the metropolitan region, Cuiabá, and Várzea Grande, as well as in smaller municipalities. These findings, allied to earlier investigations, indicate the presence of the disease in different biomes that make up the State (Queiroz et al., 2012; Thies et al., 2018; Brito et al., 2019; Menegatti et al., 2020).

Furthermore, VLCP initiatives suffered considerable impacts due to the COVID-19 pandemic. In the region of São José do Rio Preto, in the State of São Paulo, the effects of the pandemic were significant and could result in an increase in cases in the coming months or years, as activities aimed at controlling dogs and vectors were suspended and HVL cases showed a progressive decrease in notifications in 2019, 2020 and 2021, totaling 116, 76 and 41 reported cases, respectively (Bertollo & Soares, 2022).

Interestingly, our findings diverge from this pattern, as we observed the highest number of HVL cases precisely in 2020, marked by the beginning of the pandemic. It is important to highlight that treatment for HVL is exclusively provided by the Unified Health System, making the occurrence of underreporting less likely. However, we cannot rule out the possibility of underdiagnosis, given the health emergency situation established during the pandemic.

In an entomological investigation of the last triennium, the vectors *Lutzomyia longipalpis* and *Lutzomyia cruzi* were identified in 50% (8/16) of the analyzed municipalities. Note that only a few of these municipalities reported VL, indicating a potential discrepancy between vector presence and disease incidence. According to VLPC guidelines, entomological investigations are crucial not only in areas with reported cases of human and/or canine leishmaniasis but also in regions without notifications (Brasil, 2021). This approach is based on the relevance of mapping the distribution of vectors to assess the potential spread of the disease and to enable the implementation of more comprehensive and effective prevention and control strategies (Oliveira et al., 2018).

The control of VL poses a significant public health challenge. In this context, it is essential to analyze and disseminate existing control strategies to increase public awareness of the disease (Mann et al., 2021). Among the recommended approaches, educational programs with community participation have stood out in the fight against VL (Dias et al., 2021). The active participation of the community in disease prevention can reduce transmission, through measures such as the use of repellents, personal and environmental hygiene, in addition to the proper management of animals, especially dogs, the main reservoirs of VL.

In this regard, canine investigations have been a fundamental strategy in VL control (Brasil, 2021). Canine positivity can serve as an indicator of vector presence in the region. This statement is consistent with our findings, since two of the five municipalities where cases of HVL were reported for the first time also confirmed the presence of infected dogs.

According to the VLPC, investigations of canine VL are more relevant than spontaneous inquiries. In the former, all the animals are subjected to testing, enabling the detection of asymptomatic dogs, which play a crucial role as carriers of the disease in the environment (Dias et al., 2021). This approach allows one to identify infected dogs in different regions of a municipality, implement control measures and carry out proper management of affected animals.

However, our findings indicate that few municipalities followed the VLCP recommendations. Only 43 of the 141 municipalities conducted a canine investigation during the period. Of these, 20 did not register dogs with VL, which may be attributed to the low number of sampled dogs in relation to the existing canine population, resulting in underreporting of cases. This situation is described in Table 3, which highlights several municipalities with fewer than 20 canine samples collected per year. In other words, most of the municipalities did not reach the adequate sample size, since only one municipality, Poconé, reached the sample goal in the three consecutive years.

Sample size is calculated by estimating the canine population in the study area and the expected prevalence, adopting a significance level of 5%. A significance level of 2% is recommended in situations where prevalence is unknown. Additionally, according to A.O.N. Monteiro (personal communication, August 15, 2023), a veterinarian of the Superintendency of Health Surveillance, the definition of sample target per municipality may vary according to the municipality’s geographic location, sanitary status and epidemiological profile.

The data from the canine survey unequivocally demonstrate that the reality of MT is not adequately represented. This discrepancy can be attributed to insufficient adherence by municipalities and the execution of fragmented and incomplete activities. Data from 2019 indicate that, even before the COVID-19 pandemic, there were deficiencies in the implementation of the VLCP in the State. Additionally, notifications relating to the years 2020 and 2021 may have been even more impacted due to the publication by the Ministry of Health of Information Note No. 10/2020, which determined the rescheduling of serological survey actions in dogs for the post-emergency period of COVID-19, maintaining service on spontaneous demand (Brasil, 2020).

In our study, we observed a decrease in canine sample collection during the pre-pandemic period (2019) compared to the pandemic period (2020). However, it is noteworthy that there was a remarkable increase in the number of canine samples collected in 2021, still during the pandemic, both in the canine survey and through spontaneous demand.

Ensuring proper implementation of VL control actions is essential to reduce the incidence of the disease and prevent its spread. However, those responsible for implementing these measures report encountering various obstacles in the municipalities. These obstacles include the inherent complexity of controlling the disease, resistance from the population towards activities carried out by professionals from the Vec tor and Zoonosis Unit (UVZ), and the costs associated with these actions (von Zuben & Donalísio, 2016). Such operational and financial challenges can significantly compromise the effectiveness of VL control measures.

In addition, factors such as high turnover of human resources, management failures, and insufficient training of UVZ staff can negatively impact the execution of VL surveillance and other zoonosis-related actions (Furquim et al., 2022). It is worth mentioning that the effectiveness of control measures requires their continuance (Sevá et al., 2016). Considering the actions carried out by the municipalities over the three-year period, it is reasonable to assume that the State of Mato Grosso is facing these same operational and financial challenges.

Faced with the aforementioned challenges, it is essential to raise awareness among health authorities and municipal managers about the significance of VL and the need to implement effective control strategies. Appropriate investments must be allocated to human resources, continuous training, public awareness and financial support to overcome these adversities and ensure the effectiveness of VL control actions. Moreover, it is critical to promote the exchange of knowledge and experiences between municipalities, enabling the sharing of good practices and lessons learned, so that these challenges can be addressed collaboratively. Furthermore, collaboration between different sectors, including researchers, health professionals, municipal managers and the community in general, is essential to achieve significant advances in VL control and in the protection of public health.

## Conclusions

VL assumes a substantial role in MT, characterized by a continuous process of expansion and dissemination. Our investigation revealed the identification of cases of HVL in five previously unregistered municipalities, further emphasizing the expansion of the disease in the State. The limited number of municipalities in MT that adhered to the actions recommended by the VLCP in 2019, a pre-pandemic period, suggests that, even before the outbreak of the Covid-19 pandemic, a scenario of negligence in relation to the disease was already emerging. The detection of fragmented and incomplete actions in less than one-third of the municipalities over the past three years underscores the imperative need for the review and improvement of VL surveillance strategies.
